# New insights in lipid metabolism: potential therapeutic targets for the treatment of Alzheimer’s disease

**DOI:** 10.3389/fnins.2024.1430465

**Published:** 2024-09-11

**Authors:** Yuan Cao, Lin-Wei Zhao, Zi-Xin Chen, Shao-Hua Li

**Affiliations:** ^1^Department of Neurology, The First Affiliated Hospital of Zhengzhou University, Zhengzhou University, Zhengzhou, China; ^2^NHC Key Laboratory of Prevention and Treatment of Cerebrovascular Diseases, Zhengzhou, China; ^3^Clinical Systems Biology Laboratories, Translation Medicine Center, The First Affiliated Hospital of Zhengzhou University, Zhengzhou University, Zhengzhou, China; ^4^Department of Cardiology, People’s Hospital of Zhengzhou University, Henan Provincial People’s Hospital, Zhengzhou University Central China Fuwai Hospital, Zhengzhou, China

**Keywords:** lipid, lipid metabolism, Alzheimer’s disease, therapy strategies, diet

## Abstract

Alzheimer’s disease (AD) is increasingly recognized as being intertwined with the dysregulation of lipid metabolism. Lipids are a significant class of nutrients vital to all organisms, playing crucial roles in cellular structure, energy storage, and signaling. Alterations in the levels of various lipids in AD brains and dysregulation of lipid pathways and transportation have been implicated in AD pathogenesis. Clinically, evidence for a high-fat diet firmly links disrupted lipid metabolism to the pathogenesis and progression of AD, although contradictory findings warrant further exploration. In view of the significance of various lipids in brain physiology, the discovery of complex and diverse mechanisms that connect lipid metabolism with AD-related pathophysiology will bring new hope for patients with AD, underscoring the importance of lipid metabolism in AD pathophysiology, and promising targets for therapeutic intervention. Specifically, cholesterol, sphingolipids, and fatty acids have been shown to influence amyloid-beta (Aβ) accumulation and tau hyperphosphorylation, which are hallmarks of AD pathology. Recent studies have highlighted the potential therapeutic targets within lipid metabolism, such as enhancing apolipoprotein E lipidation, activating liver X receptors and retinoid X receptors, and modulating peroxisome proliferator-activated receptors. Ongoing clinical trials are investigating the efficacy of these strategies, including the use of ketogenic diets, statin therapy, and novel compounds like NE3107. The implications of these findings suggest that targeting lipid metabolism could offer new avenues for the treatment and management of AD. By concentrating on alterations in lipid metabolism within the central nervous system and their contribution to AD development, this review aims to shed light on novel research directions and treatment approaches for combating AD, offering hope for the development of more effective management strategies.

## 1 Introduction

Dementia is a worldwide emergency that affects countries with aging demographics and communities at large, with around 10 million new cases emerging globally each year (Prince et al., 2015; Anstey et al., 2017). Alzheimer’s disease (AD), the predominant type of dementia, is characterized by accumulation of amyloid plaques and neurofibrillary tangles (NFTs) in the brain. AD not only impairs the functionality of the elderly, but also imposes significant physical, psychological, and economic strain on families and society. Despite the severe and enduring consequences of AD, existing treatments fail to deliver effective relief or halt disease (Blanco-Silvente et al., 2018). Consequently, there is a pressing need for innovative AD treatments.

Exploring the mechanisms underlying the pathogenesis of AD could lead to the development of novel therapeutic approaches. Interestingly, the presence of ‘adipose inclusions’ or ‘lipoid granules’ in the brains of patients with AD has been noted as an additional neuropathological hallmark, alongside the well-known amyloid plaques and NFTs, first identified by Alois Alzheimer in 1907. Initially, this third marker was largely overlooked (Foley, 2010). However, subsequent research has consistently indicated that lipid droplets (LDs), the organelles responsible for storing neutral lipids, tend to accumulate in the brains of patients with AD (Nguyen et al., 2017; Gómez-Ramos and Asunción Morán, 2007).

Recent clinical studies have underscored the significance of lipid metabolism in AD development. Research has shown that high-fat diet (HFD) correlates with the onset of AD pathologies and cognitive deficits. This is through the activation of neuronal C/EBPβ/AEP signaling and the induction of insulin resistance, alongside inflammatory and stress responses, as observed in rodent models (Liu et al., 2022; Kothari et al., 2017). Additional research has shown that targeting pro-neuroinflammation and deficits in neurogenesis could mitigate the cognitive decline caused by HFD, suggesting that lipid metabolism is crucial for neuropathological changes in AD (Wang Q. et al., 2018). In contrast to these findings, studies on the ketogenic diet, a high-fat, low-carbohydrate regimen, highlight its neuroprotective benefits, including improved mitochondrial function and reduced inflammation and apoptosis (Rusek et al., 2019). The ability of this diet to compensate for impaired glucose metabolism by providing alternative energy sources such as ketones has sparked interest in its potential as an AD treatment (Broom et al., 2019). Furthermore, the glycation of apolipoprotein E (ApoE), which hinders the transport of essential lipids to the brain and leads to lipid shortage, may contribute to the progression of AD in its later stages. Collectively, this evidence firmly links disrupted lipid metabolism to the pathogenesis and progression of AD; however, contradictory findings warrant further exploration.

Lipids are essential nutrients for all living organisms, and play a critical role in brain physiology. The brain is the second most lipid-rich organ after the adipose tissue, comprising 10–12% of its fresh weight and over 50% of its dry weight (Naudí et al., 2015; Han, 2007). The pivotal role of various lipids in brain function has led to extensive research to uncover the intricate relationships between lipid metabolism and AD pathology, offering new avenues for treatment. Therapies targeting lipid metabolism have shown varying degrees of success against AD. This review provides clinical evidence for altered lipid metabolism in AD, explores the mechanisms linking lipid metabolism with AD etiology, and assesses the potential of targeting lipid metabolism in AD therapeutics.

## 2 Lipid metabolism in the central nervous system

**FIGURE 1:**
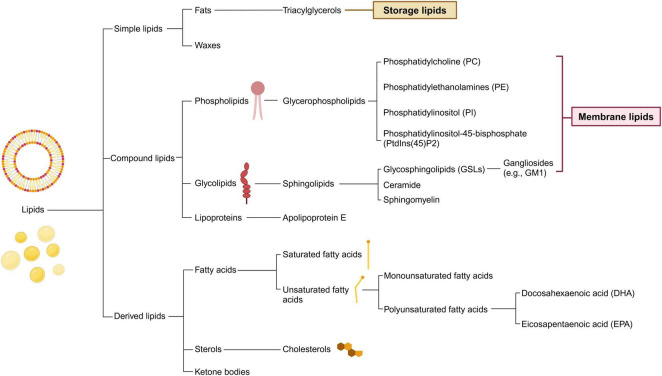
Classification of lipids and its main compounds in central nervous system. Lipids are categorized into three fundamental types: simple, compound, and derived. Within the central nervous system, the prevalent lipid species differ from those in other tissues. The flowchart illustrates common brain lipids, which are instrumental in the metabolic interchange between neurons and glial cells. Simple lipids mainly serve as storage molecules and encompass fats and waxes. Compound lipids, including phospholipids and glycolipids, are essential components of cell membranes and may contain additional functional groups. Derived lipids comprise entities such as fatty acids and sterols.

Lipids, a crucial type of macrobiomolecule, are vital for providing energy for cellular metabolism, maintaining cellular and organelle structures, and acting as signaling molecules within the central nervous system (CNS). Throughout the first 20 years of life, the levels of cerebral lipids increase, only starting to diminish after reaching 50 years of age (Naudí et al., 2015). Brain lipids predominantly fall into one of the following groups: phospholipids, sphingolipids, fatty acids (FAs), and sterols, with phospholipids accounting for approximately 50% of the brain’s total lipid content (Figure 1; Sastry, 1985). Additionally, the transport and distribution of lipids are critical for the ability of the CNS to sustain balance and support neuronal function, processes that depend significantly on apolipoproteins. Thus, understanding lipid and apolipoprotein functions is the key to understanding the fundamentals of fat metabolism (Table 1).

**TABLE 1:** Major lipid species and apolipoprotein in the central nervous system.

	Structure	Major species	Synthesis	Functions
**Lipids**				
Sterols	Composed of a four-ring core structure (cyclopentanoperhydrophenanthrene), often with a hydroxyl group at position 3 and various side chains.	Cholesterol	Endoplasmic reticulum of astrocytes and neurons. Synthesized in situ from acetyl-CoA via the mevalonate pathway, also transported from the liver via lipoproteins.	The precursor to vitamin D, testosterone, estrogen, progesterone, aldosterone, cortisol, and bile salts; structure and function of membranes; alter electrical activity in the brain. Critical for maintaining neuron membrane integrity and fluidity; precursor for neurosteroids affecting neuronal signaling.
Phospholipids	Glycerol backbone esterified with two fatty acids and a phosphate group, which may link to another alcohol, forming various subclasses.	PC, PE, PI, and PS	Endoplasmic reticulum and mitochondria of neurons, oligodendrocytes, astrocytes, and microglia. Synthesized from dietary fatty acids and glycerol via the glycerophosphate pathway or the CDP-DAG pathway.	Constituents of neuronal and glial cell membranes; involved in membrane fluidity, signaling pathways, and forming myelin sheaths.
Sphingolipids	Sphingoid base backbone, typically sphingosine, linked to a fatty acid via an amide bond, with potential for additional head groups.	Ceramide, sphingomyelin, and GSLs	Endoplasmic reticulum and Golgi apparatus of oligodendrocytes. Synthesized from serine and palmitoyl-CoA, with further modifications for specific species.	Essential for the structure of cell membranes in the CNS, especially in myelin sheaths; play roles in cell signaling and neuronal protection.
Glycerolipids	Glycerol backbone esterified with fatty acids; variations include monoglycerides, diglycerides, and triglycerides based on the number of fatty acids attached.	Triacylglycerols	Endoplasmic reticulum and mitochondria of neurons, astrocytes, oligodendrocytes. Synthesized through esterification of glycerol with three fatty acids, mainly in astrocytes.	Energy storage and provision for neurons; structural components of membranes; involved in signaling.
Fatty Acids	Long hydrocarbon chains with a carboxylic acid group at one end, varying in chain length and saturation.	Arachidonic acid and DHA	Endoplasmic reticulum and mitochondria of neurons, astrocytes, oligodendrocytes. Synthesized de novo from acetyl-CoA or modified from dietary fatty acids.	Serve as energy sources; components of phospholipids and glycerolipids in cell membranes; precursors for bioactive lipids in neuronal signaling.
**Lipoproteins and their receptors**				
ApoE	A glycoprotein with 299 amino acids; contains a receptor-binding region and a lipid-binding region.	APOE ε2, APOE ε3, and APOE ε4	Synthesized by astrocytes and to a lesser extent by microglia in the CNS.	Involved in lipid transport and injury repair in the brain. APOE ε4 is associated with increased risk of Alzheimer’s disease.
LDL receptor	A cell-surface receptor that recognizes and binds LDL particles. It is composed of several domains for ligand binding, fusion, and internalization.	LDLR and LRP1	Synthesized by neurons and glial cells.	Mediates the uptake of cholesterol-rich LDL particles for use in membrane synthesis and repair; also involved in the transport of APOE-containing lipoproteins.
HSPG	Complex molecules consisting of a protein core with one or more covalently attached heparan sulfate chains.	Syndecans and glypicans	Synthesized by a wide variety of cells in the CNS, including neurons and glial cells.	Modulates cell signaling by interacting with growth factors, morphogens, and adhesion molecules; involved in the regulation of synapse formation and neural plasticity.

ApoE, apolipoprotein E; LDL, low-density lipoprotein; HSPG, heparin sulfate proteoglycan; PC, phosphatidylcholine; PE, phosphatidylethanolamines; PI, phosphatidylinositol; PS, phosphatidylserine; GSLs, glycosphingolipids; DHA, docosahexaenoic acid; LDLR, low-density lipoprotein receptor; LRP1, LDLR-related protein 1; CoA, coenzyme A; CDP-DAG, cytidine diphosphate diacylglycerol; CNS, central nervous system.

### 2.1 Lipids

#### 2.1.1 Cholesterols

The brain, which is rich in cholesterol, contains approximately 25% of the body’s total cholesterol, which is primarily unesterified, in addition to minor amounts of desmosterol and cholesteryl ester, distinguishing it from other tissues (Dietschy and Turley, 2001; Dietschy and Turley, 2004). Cholesterol in the CNS is divided into two main pools: one found in the myelin sheaths, accounting for 70%–80% of the brain’s cholesterol, and the other in the neuronal plasma membranes, sourced from astrocytes via cholesterol-rich lipoproteins, including ApoE (Dietschy and Turley, 2004; Orth and Bellosta, 2012; Zhang and Liu, 2015). This distribution is crucial for axonal insulation, neuronal morphology, and synaptic connectivity. The brain’s cholesterol metabolism, which is vital for its function, operates independently from the rest of the body because of the blood-brain barrier (BBB), and disruptions in cholesterol homeostasis are linked to neurodegenerative diseases and cognitive decline in the elderly (Morell and Jurevics, 1996).

Cholesterol synthesis and regulation occur in the endoplasmic reticulum of astrocytes and neurons, and are managed by mechanisms contrastingly independent of and dependent on the Golgi apparatus (DeGrella and Simoni, 1982; Heino et al., 2000). This synthesis is regulated by sterol regulatory element-binding proteins and liver X receptors (LXRs), which adjust cholesterol levels through feedback loops that control its production and eflux, particularly through ATP-binding cassette (ABC) transporters (Vance et al., 1994; Barber and Raben, 2019; Moutinho and Landreth, 2017). In neurons, excess cholesterol is managed by efflux to ApoE-containing particles, maintaining cellular health and facilitating functions, such as synapse and dendrite formation, axonal guidance, and neurotransmission (Kim et al., 2008). Because cholesterol is water-insoluble, very little unbound cholesterol is detected in the cytosol, and most cholesterol exists in protein-bound forms, such as ApoE-containing cholesterol particles in the cytosol (Zhang and Liu, 2015). The role of cholesterol in neuronal physiology underscores its importance in both developmental and adult neurofunction, with its depletion leading to significant neural impairments (Linetti et al., 2010; Liu et al., 2010; Liu et al., 2007).

#### 2.1.2 Phospholipid

**FIGURE 2:**
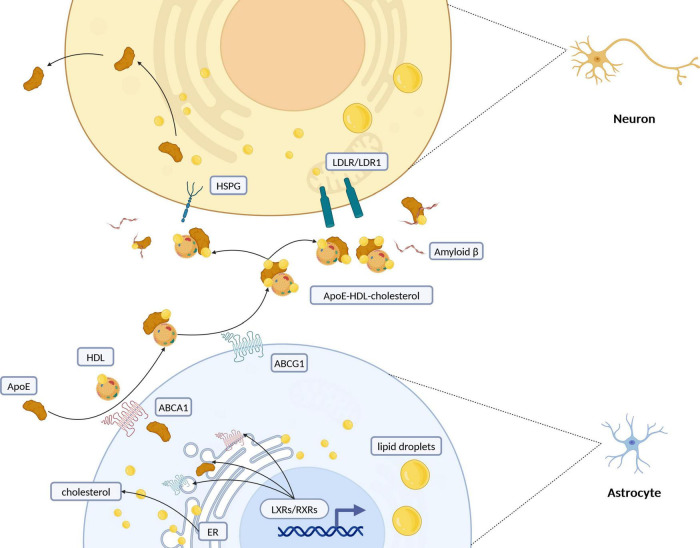
Schematic representation of intercellular cholesterol transportation between astrocytes and neurons. Cholesterol synthesis and regulation primarily take place in the endoplasmic reticulum (ER) of astrocytes. Once synthesized, cholesterol is packaged into lipid droplets and transported predominantly from glial cells to neurons. Its efflux is managed by the formation of ApoE-containing HDL-like particles, facilitated especially by ATP-binding cassette (ABC) transporters such as ABCA1 and ABCG1. Retinoid X receptors (RXRs) and liver X receptors (LXRs), part of the nuclear receptor superfamily, regulate cholesterol levels through feedback mechanisms that control its synthesis and efflux, particularly via ABC transporters. Cholesterol is redistributed to neurons through interactions with low-density lipoprotein receptors (LDLR/LDLR1) or heparan sulfate proteoglycans (HSPG). Additionally, the interaction of lipoprotein particles with amyloid beta (Aβ) peptides suggests a complex interplay between cholesterol metabolism and the pathogenesis of Alzheimer’s disease. ApoE, apolipoprotein E; HDL, high-density lipoprotein; ER, endoplasmic reticulum; RXRs, retinoid X receptors; LXRs, liver X Receptors; LDLR, low-density lipoprotein receptor; LRP1, LDLR-related protein 1; HSPG, heparin sulfate proteoglycan. Created with BioRender.com.

The predominant phospholipids in the CNS are glycerophospholipids (GPs), including phosphatidylcholine (PC) and phosphatidylethanolamines (PE), which are crucial for the cell membrane structure (Figure 2), each comprising two fatty acyl tails and a phosphate polar head on a glycerol backbone.

Studies on mild cognitive impairment (MCI) and AD subjects’ post-mortem brains have identified reduced glycerophospholipid levels in regions such as the hippocampus and cortex, which are areas susceptible to AD (Guan et al., 1999; Nitsch et al., 1992; Stokes and Hawthorne, 1987; Pettegrew et al., 2001). Research has revealed alterations in phospholipid composition and metabolism in AD, including changes in phospholipid-metabolizing enzymes (Stokes and Hawthorne, 1987; Garner, 2010). Phosphatidylinositol-4,5-bisphosphate (PtdIns(4,5)P2), which is vital for plasma membrane functions, has been linked to γ-secretase activity and amyloid beta (Aβ) 42 levels, inversely affecting AD progression (Landman et al., 2006). The hydrolysis of PtdIns(4,5)P2 by phospholipase C (PLC) increases intracellular Ca2+, implicating familial AD mutations and suggesting that PLC inhibition could reduce Aβ42 secretion and Ca2+ entry (Landman et al., 2006; Yoo et al., 2000). Furthermore, phospholipase D (PLD), particularly PLD1, plays a role in amyloidogenesis by affecting PC metabolism, influencing the cell surface delivery of amyloid precursor protein (APP) and presenilin-1 (PS1) and modulating PS1 activity, both of which are critical processes in AD development (Garner, 2010; Cai et al., 2006a; Cai et al., 2006b).

#### 2.1.3 Sphingolipids

Sphingolipids, including ceramide and sphingomyelin, and glycosphingolipids (GSLs), such as gangliosides, are crucial for the composition of lipid rafts alongside cholesterol. Ceramides play a pivotal role in sphingolipid metabolism, acting as foundational elements for creating sphingomyelin and complex GSLs by attaching to phosphocholine or sugars. These components are not only abundant in the brain, but are also essential for the structure of neuronal membranes, with gangliosides being significant for their high sialic acid content.

Studies have identified significant changes in sphingolipid levels at the onset of AD, with further results showing that increased ceramide levels that may contribute to neuronal death through oxidative stress and decreased sphingomyelin levels, implicating these lipids in the metabolism of APP and suggesting a complex role in AD progression (He et al., 2010; Castro et al., 2009; Söderberg et al., 1992). Further investigation into gangliosides reveals their significant presence in membrane rafts, affecting Aβ aggregation (Yu et al., 2011; Ariga et al., 2008). The interaction between specific gangliosides, such as GM1 gangliosidosis (GM1), and Aβ alters Aβ’s structure, facilitating the formation of amyloid fibrils and plaques associated with AD. Moreover, the manipulation of ganglioside levels, particularly through genetic modifications, has been shown to affect Aβ deposition in both the brain parenchyma and vascular tissues, illustrating the nuanced role gangliosides play in AD pathology and their potential impact on immune responses and plaque maintenance (Ariga et al., 2008; Matsuzaki, 2020; Kracun et al., 1990; Kracun et al., 1991).

#### 2.1.4 Fatty acids

Short- and medium-chain FAs are transported to the brain from peripheral sources, whereas the synthesis of long- and very-long-chain FAs occurs predominantly within the brain, starting with acetyl-CoA (Tracey et al., 2018). Moreover, FAs in the brain can also be produced through the hydrolysis of phospholipids, a process catalyzed by phospholipase A2, and possibly through the mechanism of lipophagy (Du et al., 2009; Singh and Cuervo, 2012). Astrocytes are the primary cell type involved in FAs degradation; however, microglia also contribute to this process, playing a role in determining the microglial phenotype through FA metabolism (Eraso-Pichot et al., 2018; Fecher et al., 2019; Edmond et al., 1987).

FAs are categorized as saturated, trans-, monounsaturated (MUFAs), or polyunsaturated (PUFAs), depending on their bond structures, with PUFAs being particularly significant. Despite the brain’s ability to produce saturated FAs, it relies on external sources of PUFAs acquired from peripheral blood through passive diffusion or ATP-dependent transporters (Dabravolski et al., 2021). FAs traverse the BBB either by dissociating from albumin carriers and entering endothelial cells without ATP or through transport proteins such as fatty acid transport proteins (FATP1-6), fatty acid translocase (FAT/CD36), intracellular fatty acid-binding proteins (FABP1-9), plasma membrane fatty acid binding protein (FABPpm), and caveolin-1 (Mitchell et al., 2011). These mechanisms are crucial for regulating FA metabolism in the brain. FAs are fundamental for synthesizing lipid classes and excluding cholesterol, and they play a pivotal role in forming phospholipids and sphingolipids. Notably, PUFAs, such as docosahexaenoic acid (DHA) and arachidonic acid, are vital for modulating synaptic plasticity and neurotransmission, underscoring their importance in the CNS (Bazinet and Layé, 2014; Liu et al., 2024).

### 2.2 Lipoproteins and its receptors

Mature neurons have a high demand for cholesterol, and while they can synthesize it under physiological conditions, additional ApoE-associated cholesterol is required (Pfrieger and Ungerer, 2011; Nieweg et al., 2009). ApoE, a 34 kDa glycoprotein consisting of 299 amino acids, is primarily synthesized by astrocytes within the CNS and plays a crucial role in lipid homeostasis (Mahley, 1988; Xu et al., 2006; Kang et al., 2018). With three common alleles—ε2, ε3, ε4—the isoforms of ApoE exhibit varied efficiencies in cholesterol and phospholipid binding/transporting due to structural differences. Notably, ApoE2 demonstrates superior cholesterol efflux capabilities compared to ApoE3 and ApoE4 (Minagawa et al., 2009; Wahrle et al., 2007; Hara et al., 2003).

**FIGURE 3:**
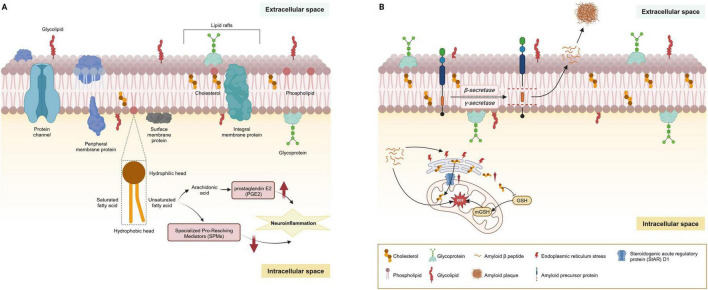
Main lipids of cell membranes and their dysregulation in AD. **(A)** Lipids are the primary constituents of cell membranes in the central nervous system. Under physiological conditions, the cell membrane is principally composed of phospholipids arranged in a bilayer. These phospholipids consist of a hydrophilic head and a hydrophobic tail; the tail may be comprised of saturated or unsaturated fatty acids, varying according to the membrane’s function and location. Additionally, cholesterol and sphingolipids are interspersed throughout the membrane, playing a significant role in the formation of lipid rafts. Glycoproteins and glycolipids are embedded in the membrane’s extracellular surface, contributing to cellular recognition and signaling. **(B)** Lipid rafts are crucial in the amyloidogenic processing of APP, where the enrichment of cholesterol and sphingolipids facilitates the anchoring of AD-related proteins such as β-site APP cleaving enzyme 1 (BACE1) and γ-secretase. The production of Aβ peptides begins with APP embedded in the cell membrane. This protein is sequentially cleaved by β-secretase and γ-secretase enzymes, releasing Aβ peptides into the extracellular space. Over time, these peptides can aggregate to form amyloid plaques. MAMs, rich in cholesterol and sphingomyelin, structurally mirror lipid rafts and are critical for lipid synthesis and trafficking. The steroidogenic acute regulatory protein (StAR) D1 is essential for mitochondrial cholesterol trafficking and is located at the MAM interface between the ER and mitochondria. ER stress induced by amyloid-β may contribute to the upregulation of StARD1. Accumulation of cholesterol in mitochondria disrupts the physical properties of the membrane and impairs the transport of GSH into mitochondria, undermining the mitochondrial antioxidant defense and leading to subsequent oxidative stress. AD, Alzheimer’s disease; AbPP, amyloid-β precursor protein; APP, amyloid precursor protein; MAMs, mitochondria-associated membranes; ER, endoplasmic reticulum.

In the brain, cholesterol transport predominantly occurs from glial cells to neurons, with ApoE acting as the main transporter by enabling cholesterol efflux from astrocytes and uptake by neurons (Figure 3A) (Boyles et al., 1989; Michikawa et al., 2000). Once ApoE has been secreted from the cells, the cell surface ABC family of transporters, ABCA1 (Nieweg et al., 2009) and ABCG1 (Karten et al., 2006), transfer cholesterol and phospholipids to nascent ApoE to form high-density lipoprotein (HDL)-like particles (Horiuchi et al., 2019). ApoE is critical in redistributing cholesterol and other lipids to neurons through binding to cell-surface ApoE receptors. The CNS’s lipid metabolism intricately involves the low-density lipoprotein receptor (LDLR) family, with numerous receptors like LDLR, LDLR-related protein 1 (LRP1), and others playing pivotal roles in the uptake of ApoE-containing lipoprotein particles (Herz, 2009; Pottier et al., 2012; Pitas et al., 1987). The distribution of these receptors—LRP1’s prevalence in neurons versus LDLR’s in glial cells—highlights the specialized functions and interactions within the brain’s lipid homeostasis framework (Rebeck et al., 1993). LRP1, known for its rapid endocytic rates, demonstrates significant transport capacity for ApoE, underscoring the dynamic interplay between ApoE isoforms, their lipidation profiles, and receptor affinities (Li et al., 2001; Fagan et al., 1996). Additionally, ApoE-enriched lipoproteins are captured by binding to heparin sulfate proteoglycan (HSPG), brought into the environment of the LRP, and then transferred to the LRP for internalization by the cells or form a complex that is internalized (Huang and Mahley, 2014). The efficacy of these processes is influenced by the lipidation extent of ApoE particles, with variations observed across different ApoE isoforms, significantly affecting lipid metabolism and receptor interaction capacities (Qi et al., 2021; Zhao et al., 2017; Lanfranco et al., 2020).

## 3 Lipid metabolism changes in AD

Lipid metabolism undergoes significant changes across the lifespan, and these changes are critical in the context of AD. For instance, cholesterol levels in the brain are known to increase with age, which can exacerbate Aβ accumulation and plaque formation, key features of AD. Moreover, the reduction in the efficiency of lipid transport mechanisms with age can lead to lipid imbalances and contribute to neurodegeneration. Sex differences also play a pivotal role in lipid metabolism and AD risk. Women generally have higher HDL cholesterol levels and differences in fatty acid composition compared to men. These differences can influence the susceptibility to and progression of AD. Estrogen, which has neuroprotective properties and modulates lipid metabolism, declines significantly in postmenopausal women, potentially increasing their risk of developing AD. Our review focuses on the intricate relationships between lipid metabolism and AD pathology. However, we acknowledge the necessity to highlight how age and sex differences contribute to these processes.

### 3.1 Cholesterol metabolism

Cholesterol levels are notably altered. Previous studies have shown that low cholesteryl esters and free cholesterol levels are associated with increased amyloid production (Abad-Rodriguez et al., 2004). However, studies in cell cultures have shown that cholesterol deficiency can increase the risk of damage from excitotoxicity (Chou et al., 2003), affect neurosteroid production (Brinton, 2013), and alter cell membrane properties. Rodent studies have shown that HFD correlates with the onset of AD pathologies and cognitive deficits through the activation of neuronal C/EBPβ/AEP signaling (Liu et al., 2022). Despite these findings, studies have generally shown that cholesterol levels in the brain and blood of AD patients are higher than those in healthy individuals, correlating with disease severity (Cutler et al., 2004; Heverin et al., 2004; Popp et al., 2013; Liu et al., 2020). Additionally, this increase has been observed in the plaque cores of AD-affected brains (Mori et al., 2001).

#### 3.1.1 Cholesterol and ApoE

Mounting evidence suggests that cholesterol levels regulate the amyloidogenic pathway (Di Paolo and Kim, 2011; Puglielli et al., 2001). Suppression of cholesterol synthesis in astrocytes substantially reduces amyloid and tau pathology in a mouse model of AD (Wang et al., 2021). Initial investigations into AD’s lipid-related mechanisms of AD have revealed disrupted cholesterol movement from astrocytes to neurons. ApoE serves as the primary cholesterol conduit, also playing a role in binding and removing Aβ peptides. Furthermore, the ApoE4 allele, a genetic variant of ApoE, is associated with AD risk, and is linked to changes in cholesterol and sphingolipids, highlighting its critical role in AD pathogenesis and lipid homeostasis (as explained in detail below) (Lane and Farlow, 2005; Bandaru et al., 2009).

#### 3.1.2 Cholesterol and lipid rafts

Lipid rafts, characterized by their dynamic nature within cell membranes, are critical for signal transduction, cell adhesion, and sorting of lipids and proteins. These structures primarily consist of sphingolipids, cholesterol, and saturated FAs, with a lower content of PUFAs. These structures are pivotal in the amyloidogenic processing of AβPP, where the enrichment of cholesterol and sphingolipids facilitates the anchoring of AD-related proteins such as β-site APP cleaving enzyme 1 (BACE1) and γ-secretase (Figure 3B). Research has underscored the role of cholesterol in lipid rafts in bringing APP and BACE1 closer, promoting rapid endocytosis, with the activities of BACE1 and γ-secretase also being influenced by cholesterol levels, indicating the significant impact of cholesterol metabolism on AD development (Allen et al., 2007; Nickels et al., 2019; Fabelo et al., 2014).

Furthermore, alterations in the composition of lipid rafts, particularly the lipid makeup, are crucial for the progression of neurodegenerative disorders such as AD. Lipid rafts serve as essential platforms for signaling pathways, and their disruption can lead to abnormal protein distribution and aggregation, thus contributing to neurodegeneration. Key components of AD pathology, including β and γ-secretase, AβPP, and Aβ oligomers, are localized within lipid rafts, exacerbating toxicity and disrupting signaling. The interaction between Aβ and ApoE in the lipid rafts is also vital for neurofibrillary tangle formation, highlighting the importance of these membrane domains in AD pathogenesis, as well as the potential of targeting cholesterol synthesis in astrocytes as a therapeutic approach to mitigate amyloid and tau pathology (Wang et al., 2021; Yin et al., 2019; Kojro et al., 2001; Lee et al., 1998; Wahrle et al., 2002; Oshima et al., 2001; Kawarabayashi et al., 2004; Riddell et al., 2001).

#### 3.1.3 Cholesterol and membrane fluidity

Cholesterol deficiency could alter cell membrane fluidity modifying their physicochemical properties. The composition of the acyl chains in membrane lipids dictates membrane fluidity, with straight and saturated acyl chains packed closely together. Integral membrane proteins such as AβPP, as well as β-and γ-secretases, rely heavily on the lipid levels within the membrane for their functionality. Cholesterol homeostasis has been highlighted as a key factor affecting amyloid production, with studies having shown its significant influence (Fabiani and Antollini, 2019; Hartmann et al., 1994). The process of AβPP cleavage by secretases is closely tied to the membrane’s physicochemical state, with increased fluidity enhancing α-secretase activity (thus preventing Aβ peptide formation) and decreased fluidity boosting γ-secretase activity, leading to amyloidogenic processing of AβPP, and an increase in longer Aβ forms (Zhang et al., 2012; Niu et al., 2018; Tan and Gleeson, 2019; Verdier et al., 2004). Additionally, cholesterol is involved in forming amyloid pores triggered by various Aβ peptides (Aβ 1–42, Aβ 22–35, Aβ 25–35) (Di Scala et al., 2013; Hashemi et al., 2022; Kandel et al., 2019).

#### 3.1.4 Cholesterol and acetylcholine receptors

The interaction of Aβ with cell membranes disrupts the structure and fluidity of the lipid microenvironment, impacting the functionality of membrane receptors, notably the α7 nicotinic acetylcholine receptor (α7 nAChR) (Small et al., 1996). Cholesterol, the principal lipid, plays a crucial role in determining the function, stability, and supramolecular organization of nAChRs within the plasma membrane. Consequently, Aβ’s detrimental effects on membrane lipids lead to a dysfunction in nAChR activity, contributing to cognitive decline (Small et al., 1996). In the CNS, neuronal nAChR, particularly α7nAChR, is closely associated with lipid rafts, and is essential for their formation and maintenance (Brusés et al., 2001; Head et al., 2014). Evidence suggests that reducing cholesterol levels decrease the presence of α7AChRs on the cell surface (Peña et al., 2011; Borroni and Barrantes, 2011), while inhibiting cholesterol synthesis alters the desensitization kinetics of α7 nAChRs without affecting their current amplitude, thereby enhancing cholinergic function and, subsequently, cognition and memory in animal models (Hernandez et al., 2010).

#### 3.1.5 Cholesterol metabolism and Aβ production

Cholesterol plays a crucial role in maintaining membrane integrity and fluidity, which are essential for the proper functioning of membrane-bound enzymes involved in Aβ production. Elevated cholesterol levels in neuronal membranes have been shown to increase the cleavage of APP by β-secretase and γ-secretase, leading to higher Aβ production (Di Paolo and Kim, 2011). Additionally, cholesterol-rich lipid rafts serve as platforms for the colocalization of APP and secretases, thereby facilitating the amyloidogenic pathway (Allen et al., 2007).

#### 3.1.6 Cholesterol and tau pathology

Cholesterol accumulation in neurons can disrupt cellular signaling pathways, leading to tau hyperphosphorylation. Elevated cholesterol levels enhance the activity of kinases such as glycogen synthase kinase-3β (GSK-3β) and cyclin-dependent kinase 5 (CDK5), which are known to phosphorylate tau (Puglielli et al., 2001). Moreover, cholesterol-induced oxidative stress can activate stress kinases, further contributing to tau pathology (Huang et al., 2001).

### 3.2 Phospholipids metabolism

Phospholipids play a pivotal role in the activities of γ-secretase and APP processing proteins (Do Carmo et al., 2024). Investigations into AD patient-derived cell cultures have uncovered shifts in various phospholipid types, such as PC, PE, and phosphatidylinositol (PI), along with enzymes, such as PLC and PLD, responsible for phospholipid metabolism (Di Paolo and Kim, 2011). Studies in animal models have indicated that changes in phospholipid composition and metabolism are linked to the pathogenesis of AD, with alterations in key enzymes affecting amyloidogenesis (Garner, 2010; Cai et al., 2006b). GPs, crucial structural components of cellular membranes, influence membrane fluidity and have been found to be dysregulated in AD, showing a widespread reduction across PC, PE, and PI species (Bennett et al., 2013; Kosicek and Hecimovic, 2013; Wong et al., 2017). Moreover, changes in plasmalogen PE levels in AD brains could act as potential lipid biomarkers, with a significant decrease in white matter and an increase in gray matter developing as AD progresses (Han et al., 2001). PtdIns(4,5)P2, a key derivative of PI and a substrate for PLC, sees reduced turnover upon PLC inhibition, which subsequently lowers Aβ42 secretion (Landman et al., 2006). Conversely, inhibiting PLC activity adversely affects the activity of non-amyloidogenic γ-secretase (Rossner, 2004), casting doubt on PLC signaling’s role in mitigating amyloidogenesis in AD. Collectively, the correlation between phospholipid metabolism and APP processing is further underscored by the accelerated intracellular Aβ accumulation and memory issues stemming from a phospholipid transfer protein deficiency (Tong et al., 2015).

### 3.3 Sphingolipid metabolism

Research on cell cultures has shown that Aβ42 and Aβ40 can reduce sphingomyelin and cholesterol levels by activating neutral sphingomyelinases and inhibiting hydroxymethylglutaryl-CoA reductase activity (Grimm et al., 2005). In rodent models, increased ceramide levels contribute to neuronal death through oxidative stress, implicating sphingolipid metabolism in AD progression (He et al., 2010). Analyses of AD patient brains have shown elevated levels of acid sphingomyelinase and acid ceramidase, leading to decreased sphingomyelin levels and increased ceramide production, which in turn reduces γ-secretase activity and Aβ secretion (He et al., 2010; Grimm et al., 2005; Di Paolo and Kim, 2011). This interaction showcases the close relationship between Aβ generation and the metabolism of cholesterol and sphingolipids. Interestingly, Aβ42 and Aβ40 can further diminish sphingomyelin and cholesterol levels by activating neutral sphingomyelinases and inhibiting hydroxymethylglutaryl-CoA reductase activity, respectively, suggesting a reciprocal regulatory mechanism (Grimm et al., 2005). Moreover, fibrillar Aβ triggers sphingomyelinase activity and ceramide production through ROS-dependent pathways, potentially creating a feedback loop that amplifies ceramide accumulation and worsens AD pathology (Grimm et al., 2005; Jana and Pahan, 2004; Malaplate-Armand et al., 2006).

Mitochondria-associated membranes (MAMs), critical for lipid synthesis and trafficking, mirror lipid rafts structurally and function as a nexus for lipid and Aβ metabolism within neurons (Figure 3B; Sezgin et al., 2017; Vance, 2014; Giorgi et al., 2015). MAMs are rich in cholesterol and sphingomyelin, which are house-keeping proteins essential for phospholipid exchange, calcium signaling, and redox regulation. Studies have revealed an enrichment of β- and γ-secretases at MAMs, linking the elevated β-secretase cleaved APP product (C99) to diminished mitochondrial function, spurred by increased sphingomyelin hydrolysis and ceramide levels (Pera et al., 2017; Area-Gomez et al., 2009; Area-Gomez et al., 2018). Ceramides play a role in stabilizing BACE1, extending its half-life, and thus elevating the Aβ production rates (Puglielli et al., 2003). GSLs are also implicated in amyloid fibril formation, with the GM1 specifically binding to Aβ. These GM1-Aβ complexes, identified in early-stage AD patient brains, are linked to the presence of Aβ oligomers in the cerebrospinal fluid (CSF), underlining the significant impact of sphingolipids on APP processing, Aβ generation, and amyloid accumulation (Hong et al., 2014). This interplay between sphingolipids and Aβ suggests a critical role for these lipids in the molecular mechanisms underlying AD progression.

### 3.4 Fatty acids metabolism

The cortex of individuals with AD reveals increased FAs synthase protein levels and elevated free fatty acids (FFAs), which disrupt mitochondrial function and energy metabolism (Ates et al., 2020; Thangavel et al., 2017; Schönfeld and Reiser, 2017; Li L. O. et al., 2010). These compounds are neurotoxic and disrupt mitochondrial function and energy metabolism. Research has shown that the levels of FFAs and even-chain saturated FAs are higher in the CSF of patients with AD, and that these FFAs promote the assembly of amyloid and tau filaments in vitro. Conversely, levels of unsaturated FAs, including ω-3 PUFAs, DHA, and MUFAs, are lower in the AD brain and plasma; (Fonteh et al., 2014; Cunnane et al., 2012; Snowden et al., 2017; Belkouch et al., 2016; Fonteh et al., 2020). The lipid raft profiles in AD brains further reflect lipid imbalances, showcasing reduced ω-3 PUFAs and MUFAs, particularly oleic acid, and a lower unsaturation index of phospholipid acyl chains, indicating alterations in lipid homeostasis from the early stages of AD (Martín et al., 2010; Marin et al., 2016; Fabelo et al., 2014).

Imbalances in fatty acid metabolism, particularly an increase in saturated fatty acids (SFAs) and a decrease in PUFAs, can exacerbate Aβ toxicity. SFAs are known to increase the rigidity of cell membranes, promoting Aβ aggregation, while PUFAs, such as DHA, have been shown to inhibit Aβ fibrillogenesis and enhance its clearance (Bazinet and Layé, 2014).

The alteration of fatty acid composition in neuronal membranes can influence tau phosphorylation and aggregation. For instance, the depletion of DHA has been associated with increased tau hyperphosphorylation through the activation of GSK-3β and CDK5 (Rankin et al., 2007). Conversely, supplementation with omega-3 FAs has been shown to reduce tau pathology in animal models of AD (Morris et al., 2003).

Studies in vitro have highlighted that ApoE4 impedes FA sequestration in LDs, leading to increased FFA levels and neuroenergetic and synaptic disturbances (Qi et al., 2021). The degradation of FFAs, particularly those which are oxidized, is vital for brain health, with neurons depending on glial cells, mainly astrocytes, for the clearance and degradation of excessive FFAs in LDs through oxidative processes like β-oxidation within mitochondria and peroxisomes (Schönfeld and Reiser, 2017; Ioannou et al., 2019; Qi et al., 2021).

LDs serve as an initial defense against the lipotoxicity caused by FFAs or cholesterol, trapping them as neutral triacylglycerols (TAGs) and cholesterol esters (CEs) (Nguyen et al., 2017). The accumulation of LDs in AD corresponds to their role in the presence of peroxidised FAs and susceptible PUFAs (Zhang et al., 2021). Research in rodent models has indicated that LDs accumulation in ependymal cells preludes amyloid and tau pathology, thereby impacting neural stem cell proliferation (Hamilton et al., 2015). Furthermore, in vitro evidence has highlighted that ApoE4 impedes FA sequestration in LDs, leading to increased FFA levels, and neuroenergetic and synaptic disturbances (Qi et al., 2021). During oxidative stress, peroxidized FAs move from neurons to astrocytes through ApoE-mediated processes, and ApoE4 negatively affects this transfer (Ioannou et al., 2019; Liu L. et al., 2017). Studies have revealed that while ApoE3 promotes astrocytic clearance of neuronal LDs, ApoE4 does not, underscoring the varying influence of ApoE isoforms on lipid management. Additionally, AD risk genes, such as *ABCA1*, *ABCA7*, and *PICALM*, play roles in glial LDs formation (Moulton et al., 2021), suggesting that impaired lipid clearance and transport contribute to lipid imbalance and diminished neurotrophic support in AD and aging.

### 3.5 Lipid droplets and AD pathogenesis

LDs are dynamic organelles involved in the storage and metabolism of lipids and have been increasingly recognized for their roles in cellular homeostasis and AD processes. Recent studies have highlighted several functional roles of LDs in the context of AD.

LDs store excess FAs and cholesterol, preventing lipotoxicity and maintaining cellular lipid homeostasis. This detoxification role is crucial in the AD brain, where lipid metabolism is disrupted. Studies have shown that LDs trap harmful substances as neutral TAGs and CEs to mitigate damage (Nguyen et al., 2017).

LDs provide a reservoir of lipids that can be mobilized for energy production, particularly in conditions of metabolic stress. The dysregulation of this function in AD could contribute to the observed energy deficits in affected neurons. Evidence from rodent models indicates that LDs accumulation in ependymal cells precedes amyloid and tau pathology, impacting neural stem cell proliferation and highlighting the potential link between LDs and neurogenesis (Hamilton et al., 2015).

LDs are involved in the cellular response to oxidative stress by sequestering peroxidized lipids, thus protecting cells from oxidative damage. In AD, impaired LD function due to ApoE4 can exacerbate oxidative stress and neuronal injury. Clinical studies have highlighted that LDs in neurons and glial cells are associated with the presence of peroxidized FAs and susceptible PUFAs (Zhang et al., 2021).

LDs interact with immune cells in the brain, influencing the inflammatory response. Changes in LD dynamics can affect neuroinflammation, a key feature of AD pathology. Research has shown that LDs play a role in the presence of peroxidised FAs and susceptible PUFAs, and ApoE-mediated processes are crucial for the transfer of these FAs. However, the ApoE4 isoform negatively affects this transfer, leading to inefficient lipid management and increased neuronal damage (Ioannou et al., 2019).

### 3.6 Link between lipids and mitochondria in AD

The link between lipid metabolism and mitochondrial function is crucial in understanding the pathogenesis of AD. Mitochondria are vital for cellular energy production and lipid metabolism, and their dysfunction is a hallmark of AD. Several studies have demonstrated how lipid imbalances can affect mitochondrial function, contributing to neurodegeneration in AD. MAMs are specialized subdomains of the endoplasmic reticulum (ER) that physically and functionally interact with mitochondria. MAMs are critical for lipid synthesis and trafficking, calcium signaling, and apoptosis regulation. In AD, alterations in MAMs contribute to disrupted lipid metabolism and mitochondrial dysfunction. Studies have shown that β- and γ-secretases, involved in APP processing enriched in MAMs, link lipid metabolism directly to amyloidogenic pathways (Area-Gomez et al., 2018).

Elevated levels of certain lipids, such as ceramides and cholesterol, have been shown to impair mitochondrial function. Ceramides, in particular, can induce mitochondrial apoptosis pathways, leading to neuronal death. Increased ceramide levels have been observed in AD brains, correlating with mitochondrial dysfunction and neurodegeneration (He et al., 2010).

FAs are metabolized in mitochondria through β-oxidation, producing energy. However, imbalances in FA metabolism can lead to mitochondrial stress and dysfunction. In AD, dysregulated FA metabolism results in the accumulation of toxic lipid intermediates, which impair mitochondrial respiration and increase oxidative stress. Studies have found that AD patients exhibit altered levels of free fatty acids (FFAs) and their metabolic by-products, which are neurotoxic and disrupt mitochondrial function (Ates et al., 2020).

Mitochondrial dysfunction in AD is often accompanied by increased oxidative stress and lipid peroxidation. Oxidative damage to lipids within mitochondrial membranes can further impair mitochondrial function, creating a vicious cycle of mitochondrial dysfunction and lipid peroxidation. This oxidative stress is exacerbated in AD, leading to progressive neuronal damage (Song et al., 2021).

### 3.7 Lipids and vascular contributions to dementia

Atherosclerosis, characterized by the buildup of cholesterol-rich plaques in the arterial walls, is a major vascular condition linked to lipid metabolism (Mourino-Alvarez et al., 2024). It leads to reduced blood flow and increased risk of cerebrovascular events such as strokes, which are significant risk factors for cognitive decline and dementia. Studies have shown that high LDL cholesterol and low HDL cholesterol levels are associated with increased risk of atherosclerosis and subsequent cognitive impairment (Poliakova and Wellington, 2023). However, controversial results from older adults showed that very high HDL-C levels were associated with an increased risk of cognitive impairment, indicating that the very high levels of HDL-C may change its protective role in maintaining brain health (Huang et al., 2024).

Cerebral Small Vessel Disease (CSVD) encompasses a range of pathological processes affecting small blood vessels in the brain, including arteriolosclerosis, microinfarcts, and microbleeds. Dyslipidemia, particularly elevated triglycerides and low HDL cholesterol, has been implicated in the development of CSVD. CSVD is a common cause of vascular cognitive impairment and can exacerbate AD pathology by promoting amyloid deposition and tau pathology (Hong et al., 2024).

### 3.8 BBB dysfunction

The BBB plays an essential role in separating the brain from and connecting the brain to peripheral circulation by forming an interface for the transcytotic exchange of substances, including lipids (Segarra et al., 2021). BBB dysfunction manifests in the early stages of AD, irrespective of changes in Aβ or tau biomarkers (Montagne et al., 2017; Nation et al., 2019). In another study focusing on the effect of the peripheral lipid profile, obesity-associated high circulating saturated FAs led to elevated CSF levels of palmitate and increased BBB permeability, and high brain levels of palmitate further elicited neuroinflammation and impaired synaptic and cognitive function (Rhea et al., 2017; Ouyang et al., 2014; Melo et al., 2020). Mfsd2a, a phospholipid flippase, and transporters expressed by BBB endothelial cells suppress transcytosis and maintain BBB integrity (Ben-Zvi et al., 2014). It has further been discovered that, as a major DHA transporter across the BBB, Mfsd2a increases DHA-containing phospholipids in CNS endothelial cells, which inhibits caveolae-mediated transcytosis and helps to maintain BBB integrity (Andreone et al., 2017).

### 3.9 ApoE4

#### 3.9.1 ApoE4 impairs Aβ accumulation and clearance

The role of ApoE role in AD pathology is multifaceted, with evidence showing its co-deposition with Aβ in amyloid plaques (Namba et al., 1991). Post-mortem studies have further identified an increase in Aβ plaque deposition and an earlier onset of amyloid pathology in ApoE4 carriers (Kok et al., 2009; Schmechel et al., 1993), with apoE4 specifically accelerating early amyloid seeding due to its higher affinity for Aβ compared to other isoforms. Research involving AD mouse models and human-induced pluripotent stem cells has demonstrated that the apoE4 isoform not only increases Aβ load in the brain interstitial fluid, but also shows distinctive cellular behaviors, with neurons exhibiting increased synapse numbers and Aβ42 secretion, while astrocytes display reduced Aβ uptake and increased cholesterol accumulation, highlighting the isoform’s unique impact on amyloid pathology (Liu C. C. et al., 2017; Huynh et al., 2017; Lin et al., 2018).

Furthermore, while ApoE plays a critical role in Aβ clearance through mechanisms like receptor-mediated clearance and proteolytic degradation, apoE4’s effectiveness in these processes is lower than that of apoE2 and apoE3. The inefficiency of apoE4 in lipid transport extends to compromised proteolytic degradation of Aβ, leading to slower Aβ clearance. This is evidenced by in vivo studies showing differential clearance rates of ApoE-Aβ complexes by the very low density lipoproteins receptor and LRP1 on the BBB, with apoE4 complexes clearing more slowly. Additionally, ApoE4 microglia-like cells show altered morphologies and reduced Aβ phagocytosis. The interaction between ApoE and Aβ, particularly the influence of ApoE lipidation on this process, sheds light on how disruptions in lipid homeostasis may contribute to AD progression, with delipidated apoE4 particularly prone to accelerating fibril formation (Lin et al., 2018; Wildsmith et al., 2013; Sanan et al., 1994; Pillot et al., 1999).

#### 3.9.2 ApoE4 stimulates tau phosphorylation

Research has shown that fragments of apoE4 lacking parts of their carboxyl terminus can penetrate the cytosol, leading to neurotoxic effects (Pillot et al., 1999; Harris et al., 2003; Huang et al., 2001). These fragments specifically target components of the cytoskeleton such as tau and neurofilaments, thus altering their functions. In transgenic mice, these truncated ApoE4 fragments have been observed to promote tau phosphorylation and the formation of intracellular NFTs, similar to those observed in AD (Brecht et al., 2004; Harris et al., 2003). Remarkably, the removal of tau from mice offers protection against neurotoxic effects induced by these ApoE4 fragments (Andrews-Zwilling et al., 2010). Furthermore, studies in humans have identified that ApoE4 can independently increase the levels of phosphorylated tau in the CSF without the involvement of Aβ (Cruchaga et al., 2013).

#### 3.9.3 ApoE4 fragments impair mitochondrial function

Mitochondrial dysfunction in AD is influenced by the ApoE genotype, with the effects being notably more pronounced in ApoE4 carriers than in ApoE3 (Ghosh et al., 1999; Hirai et al., 2001; Kamino et al., 2000; Gibson et al., 2000). ApoE4’s association with reduced cerebral glucose metabolism has been observed in both AD patients and non-demented individuals of the same age, a phenomenon that precedes cognitive symptoms by decades and likely occurs before extensive Aβ accumulation (Hirono et al., 2002; Mosconi et al., 2004; Reiman et al., 2004; Reiman et al., 2005; Small, 2001; Small et al., 2004; Scarmeas et al., 2005). This suggests that apoE4-related mitochondrial dysfunction may be an early event in the pathogenesis of AD. Additionally, apoE4 fragments specifically interact with neuronal mitochondria, exacerbating mitochondrial dysfunction, and contributing to neurotoxicity (Chang et al., 2005). The detrimental impact of ApoE on mitochondrial dynamics is isoform-dependent, with cultured neuronal cells showing significantly reduced mitochondrial mobility in the presence of apoE4 fragments, followed by apoE4, and to a lesser extent, apoE3 (Brodbeck et al., 2011). Furthermore, in transgenic mice with neuron-specific expression of ApoE4, the axonal transport of mitochondria is impaired, highlighting the detrimental role of apoE4 in neuronal health and function (Tesseur et al., 2000).

### 3.10 Brain-derived extracellular vesicles (BDEVs)

Extracellular vesicles (EVs) are released by all the cells, and contain a subset of their parental cells, including lipids. One prior in-depth lipidomic analysis revealed that the lipid composition of brain-derived EVs (BDEVs) in the human frontal cortex is altered in AD (Su et al., 2021). AD BDEVs had significantly altered glycerophospholipid and sphingolipid levels, specifically increased PS lipids, decreased polyunsaturated fatty acyl-containing lipids, and altered amide-linked acyl chain content in sphingomyelin and ceramide lipids relative to the controls. PS lipids constitute a substantial proportion of the EV membrane and have been proposed to play a role in facilitating EVs uptake by recipient cells (Kastelowitz and Yin, 2014; Laulagnier et al., 2004; Matsumura et al., 2019; Record et al., 2014; Record et al., 2018; Sharma et al., 2017; Wei et al., 2016). The most prominent alteration was a two-fold decrease in lipid species containing anti-inflammatory/pro-resolving DHA.

Notably, BDEVs can be considered as ideal lipid peripheral biomarkers because they can readily cross the BBB (Kanninen et al., 2016; Skoumalová et al., 2011). Future studies should assess PE lipids and polyunsaturated fatty acyl-containing lipids in peripherally sourced BDEVs, alongside other clinical measures (CSF and neuroimaging assessments), to determine whether BDEVs in the periphery can predict the progression from mild MCI to AD.

### 3.11 Genetic risk factors involved in lipid metabolism

Among the key risk genes for sporadic late-onset AD, *APOE, TREM2, APOJ, PICALM, SREBP-2, ABCA1*, and *ABCA7* are involved in lipid trafficking and metabolism (Table 2; von Maydell et al., 2024). Similarly, genes linked to familial AD, including *APP*, *PSEN1*, and *PSEN2*, influence lipid metabolism (Table 2; Karch and Goate, 2015). The *APOE4* allele is identified as the most potent genetic risk factor for AD, enhancing the disease’s likelihood by 4 to 14 times depending on the presence of one or two alleles, respectively, when compared to the more common ε3 isoform. This risk alteration is attributed to a combination of the gain of toxic effects and the loss of protective functions seen with *APOE4*, whereas the ε2 isoform (*APOE2*) is associated with a reduced risk (Liu et al., 2013; Corder et al., 1994; Reiman et al., 2020). Additionally, *TREM2*, primarily located on microglial surfaces, plays a crucial role in phagocytosis and the inflammatory response, with loss-of-function mutations resulting in early onset dementia, including Nasu-Hakola disease (Jay et al., 2017; Ulland and Colonna, 2018; Kleinberger et al., 2014; Paloneva et al., 2002). Recent studies have underscored *TREM2*’s involvement in myelin debris clearance and remyelination, highlighting how *TREM2* deficiency leads to disrupted lipid metabolism and transport (Gouna et al., 2021; Nugent et al., 2020; Li et al., 2022). *APOJ* (Clusterin) and *PICALM* have also been recognized for their roles in AD risk, with *APOJ* implicated in Aβ aggregation and clearance and *PICALM* in lipid particle internalization and transport (Harold et al., 2009; Lambert et al., 2009; Zhao et al., 2015). *SREBP-2*, regulating cholesterol synthesis and has been linked to AD progression (Chan et al., 2018). These findings highlight the complex interactions between lipid-related genes and AD pathology, and further emphasize the significance of lipid metabolism and trafficking in disease development and progression (Wang Y. et al., 2015; Yeh et al., 2016; Kober and Brett, 2017; Nuutinen et al., 2009). ABCA1 and ABCA7 belong to the ABC transporter family, showing 54% sequence similarity (Aikawa et al., 2018). ABCA1 plays a crucial role in lipid efflux, specifically of cholesterol and phospholipids, by attaching them to lipid-free lipoproteins, notably ApoE, which serves as the main lipidation substrate in the brain (Nordestgaard et al., 2015; Wahrle et al., 2004). Mutations leading to ABCA1 dysfunction are linked to reduced plasma ApoE levels and increased risk of AD (Nordestgaard et al., 2015). Similarly, ABCA7 participates in the transport of cholesterol and phospholipids (Abe-Dohmae et al., 2004). Human genetic and epigenetic studies have identified various SNPs, gene variants, alternative splicing events, and methylation events in the *ABCA7* gene, all of which are associated with functional impairment. These alterations in ABCA7 have been implicated in disrupted lipid and Aβ metabolism, ultimately contributing to an increased likelihood of AD development (Aikawa et al., 2018; De Roeck et al., 2019). This study identified differentially expressed lipid metabolism-related genes (DELMRGs) in AD, emphasizing the role of these genes in disease progression and potential therapeutic targets (Zeng et al., 2023).

**TABLE 2:** Confirmed Alzheimer’s disease risk gene from large cohort genetic studies.

Gene Symbol	Function in Lipid Metabolism	Implication in AD	Reference (PMID)
*APOE*	Apolipoprotein E is involved in lipid transport and clearance.	*APOE* ε*4* allele is the strongest known risk factor for sporadic AD, affecting cholesterol metabolism and aggregation of Aβ, while *ε2* may be protective.	Corder et al., 1993; Kunkle et al., 2019
*TREM2*	Involved in the regulation of microglial function and the phagocytosis of lipids and apoptotic cells.	Mutations in *TREM2* can lead to early-onset dementia and altered lipid metabolism. *TREM2* variants may impair microglial response to amyloid plaques, leading to neuroinflammation.	Jonsson et al., 2013; Bellenguez et al., 2022
*ABCA7*	ATP-binding cassette transporter A7 is involved in lipid trafficking and metabolism.	Variants in *ABCA7* are associated with an increased risk of AD, potentially through disrupted clearance of Aβ or altered lipid metabolism.	Hollingworth et al., 2011; Bellenguez et al., 2022
*CLU (ApoJ)*	Clusterin is involved in lipid transport and clearance of cellular debris.	Variants have been associated with altered lipid metabolism and increased risk of AD.	Lambert et al., 2009; Bellenguez et al., 2022
*SORL1*	Sortilin-related receptor, involved in trafficking of APP and lipoproteins.	Variants have been linked to altered lipid processing and APP trafficking.	Rogaeva et al., 2007; Bellenguez et al., 2022
*CR1*	Modulates complement system and lipid metabolism	Variants in CR1, which plays a role in the immune response, have been linked to increased increased amyloid plaque burden and neuroinflammation.	Lambert et al., 2009; Bellenguez et al., 2022
*PICALM*	Phosphatidylinositol binding clathrin assembly protein, involved in clathrin-mediated endocytosis.	Variants affect intracellular trafficking of lipids and APP, influencing amyloid-beta accumulation.	Lambert et al., 2009; Bellenguez et al., 2022
*BIN1*	Involved in clathrin-mediated endocytosis and membrane curvature	Variants are linked to tau pathology and neuroinflammation, contributing to AD.	Lambert et al., 2013; Bellenguez et al., 2022
*MS4A6A*	Involved in immune response and lipid rafts in cell membranes	Variants are associated with altered immune response and increased AD risk.	Hollingworth et al., 2011; Bellenguez et al., 2022

HDL, high-density lipoprotein; ATP, adenosine triphosphate; APP, amyloid precursor protein; AD, Alzheimer’s disease; Aβ, amyloid-beta.

### 3.12 Immunomodulatory properties of lipid metabolism

Lipid metabolism directly influences neuroinflammation through the production of specialized pro-resolution mediators (SPMs) such as resolvins, lipoxins, protectins, and maresins, derived from PUFAs (ω-3 PUFAs, like DHA and EPA) (Basil and Levy, 2016). SPMs play crucial roles in dampening inflammation and promoting homeostasis (Emre et al., 2020; Zhu et al., 2016). In AD, neuroinflammation is a critical aspect of the pathogenesis and progression, with decreased levels of multiple SPMs observed in human AD brains, underlying the challenge of overcoming persistent inflammation. Notably, there was a positive relationship between CSF levels of SPMs (LXA4 and RvD1) and cognitive performance in both healthy and AD subjects. Experimental treatments involving SPM supplementation have been shown to counteract declines in hippocampal SPM levels, diminish inflammatory cytokines, reduce glial activation, and mitigate Aβ and tau pathology in AD mouse models (Weiner et al., 2015; Kantarci et al., 2018; Dunn et al., 2015).

Furthermore, the interplay between lipid metabolism and neuroinflammation extends to the expression of anti-inflammatory genes triggered by lipid-sensing nuclear receptors such as LXRs and PPARs (Kang and Rivest, 2012; Heneka et al., 2011). Pro-inflammatory prostaglandins, especially prostaglandin E2 (PGE2), synthesized from arachidonic acid by cyclooxygenase enzymes (COX-1 and COX-2) and prostaglandin E synthases, are found at increased concentrations in the early stages of AD, and their presence has been linked to intensified neuroinflammation, oxidative stress, and memory deficits in rodent models (Ricciotti and FitzGerald, 2011). Targeting PGE2 receptors can alleviate these adverse effects via microglial-driven mechanisms. In addition, PGE2 has been shown to impair microglial function and mitochondrial energy production via the EP2 receptor (Montine et al., 1999; Combrinck et al., 2006; Shi et al., 2012; Liang et al., 2005; Johansson et al., 2015; Minhas et al., 2021). Finally, the role of lipid rafts in glial cells, which are rich in cholesterol and sphingolipids, suggests a pivotal role in initiating inflammatory responses, where elevated cholesterol levels contribute to the aggregation of inflammatory proteins and increase inflammation (Miller et al., 2020).

## 4 Treatment of AD by targeting lipid metabolism

### 4.1 ApoE lipidation

For ApoE to carry out its essential roles, including lipid/cholesterol transport, synapse regeneration, immune modulation, and Aβ clearance/degradation, it must be secreted and adequately lipidated (Kanekiyo et al., 2014; Hanson et al., 2013). However, ApoE4 exhibits poor lipidation, and is less abundantly expressed than ApoE2 and ApoE3 (Kanekiyo et al., 2014; Wang L. et al., 2015; Heinsinger et al., 2016). This shortfall in ApoE4 lipidation indicates that enhancing ApoE lipidation may be a promising therapeutic strategy for treating AD and other neurological conditions (Table 3).

**TABLE 3:** Therapies of Alzheimer’s disease by targeting lipid metabolism.

Therapeutic Approach	Target/Method	Potential Effects on AD
ApoE Targeting		
ApoE4 Structure Correctors	Small molecules or peptides that alter the structure of the ApoE4 isoform to resemble the less pathogenic ApoE3	Potentially reduce its negative impact on lipid transport and Aβ aggregation.
ApoE Mimetics	Peptides designed to mimic the beneficial properties of ApoE	Promote lipid transport and clearance of Aβ.
**Cholesterol Management and Lipid-sensitive Nuclear Receptors**		
Statin Treatment	Reduction of cholesterol in brain membranes; anti-inflammatory.	May prevent AD progression by improving cardiovascular health and reducing vascular inflammation; potential benefits in ApoE4 carriers.
Nuclear Receptor Agonists	Targeting LXR/RXR/PPAR pathways	Might improve lipidation and decrease lipid-free availability, though peripheral side effects on liver and metabolic organs need consideration.
LXR Agonists	Compounds that activate LXR	Promote cholesterol efflux from cells, modulate inflammation, and potentially influence Aβ clearance.
RXR Agonists	Compounds that promote RXR activity	It may enhance cholesterol and lipid homeostasis, influencing processes related to AD development.
PPAR Agonists	Activation of PPAR	It can influence lipid metabolism, inflammation, and insulin sensitivity, all of which are relevant to AD pathology.
**Sphingolipid Metabolism Modulation**		
S1P Receptor Modulators	S1P signaling	S1P signaling is involved in various cellular processes, including inflammation and cell survival. Modulating this pathway could impact AD pathology.
Ceramide Reduction Strategies	Inhibitors of ceramide synthesis	It potentially reduces the levels of this sphingolipid, which is associated with increased Aβ production and neurodegeneration.
Diet Interventions		
Ketogenic Diet	High-fat, low-carbohydrate diet producing ketone bodies	Attenuating Aβ pathology improves brain metabolic and cognitive function and may act as an alternative fuel in the AD brain.
PUFAs Supplementation (e.g. Omega-3 Fatty Acids)	Increasing intake of ω-3 PUFAs, particularly DHA and EPA	Reduces Aβ production, modulates brain inflammation, improves insulin sensitivity, and potentially lowers AD risk.
Probiotic Supplementation	Manipulation of gut microbiota	Improved glucose metabolism, reduced inflammation and oxidative stress, decreased Aβ and tau aggregates, and improved cognitive functions.

ApoE, Apolipoprotein E; LXR: Liver X Receptor; RXR, retinoid X receptor; PPAR, peroxisome proliferator-activated receptor SIP, Sphingosine-1-Phosphate; PUFAs, polyunsaturated fatty acids; DHA, docosahexaenoic acid; EPA, eicosapentaenoic acid; Aβ, amyloid-beta; AD, Alzheimer’s disease.

#### 4.1.1 Small molecules that enhance ABCA1-mediated ApoE4 lipidation

Targeting ApoE lipidation has emerged as a potential strategy for mitigating AD symptoms. The ABCA1 transporter plays a crucial role in this process by transferring cholesterol to apolipoproteins, including apoE, thus regulating lipidation in the brain (Koldamova et al., 2014). Enhancing ABCA1 transporter activity can be achieved through micro-RNA manipulation, notably by inhibiting miR-33, a brain-pervasive microRNA that influences Aβ levels via ABCA1. Inhibition of miR-33 in the brain leads to increased ABCA1 and ApoE lipidation and facilitates the degradation of extracellular Aβ, simultaneously decreasing Aβ secretion in the cortex of APP/PS1 mice (Kim et al., 2015). Furthermore, the activation of ABCA1 can also be accomplished through the application of therapeutic peptides, such as CS-6253, which is derived from the C-terminal domain of ApoE. This peptide can oligomerize ABCA1, boost its activity, reverse cognitive deficits linked to ApoE4, and reduce both tau hyperphosphorylation and Aβ buildup in hippocampal neurons (Boehm-Cagan et al., 2016; Handattu et al., 2013). Another promising peptide, Ac-hE18A-NH2, counteracts the adverse effects of Aβ on ApoE secretion and, when administered to mice, enhances cognition, reduces amyloid plaques, and diminishes glial activation while increasing CNS levels of ApoE (Handattu et al., 2013). Peptide 4F, which mimics HDL function, significantly boosts ApoE secretion and lipidation in glial cells without causing cytotoxicity (Chernick et al., 2018). While these interventions primarily focus on Aβ pathology, their impact on lipid metabolism and trafficking warrants further exploration.

#### 4.1.2 ApoE4 structure correctors

Efforts to counteract the ApoE4-associated neuropathology have led to the development of structural correctors that transform ApoE4 into a more ApoE3-like conformation, enhancing lipidation and improving phospholipid and cholesterol transport (Mahley, 2016). The compound PH002 has been identified to mitigate ApoE4 toxicity in human iPSC-derived neurons, evidenced by a reduction in toxic ApoE4 fragments, an increase in GABAergic neurons and GAD67 levels, alongside a decrease in hyperphosphorylated tau and Aβ production (Wang C. et al., 2018). Additionally, compounds such as GIND-25 and GIND105 have been designed to adjust ApoE4 towards ApoE3-like lipid-binding characteristics, positively affecting neuronal cultures (Chen et al., 2011; Chen et al., 2012; Brodbeck et al., 2011). Another approach, anti-ApoE4 immunotherapy, uses antibodies targeting non-lipidated ApoE4 to reduce its toxicity. The anti-human ApoE antibody HAE-4, which identifies non-lipidated ApoE3 and ApoE4, has shown promise in reducing Aβ deposition in APP/PS1–21/ApoE4 mice, whether administered centrally or peripherally (Liao et al., 2018).

Enhancing ApoE lipidation through the activation of ABC transporters, such as ABCA1, has shown promise in preclinical studies. Further strategies include silencing of ARF6 with siRNAs to boost ABCA1 activity, as ApoE4 astrocytes express more ARF6 than ApoE3, leading to decreased ABCA1 expression on the membrane and increased degradation (Rawat et al., 2019). Advancements in genetic therapies, such as using *AAV-APOE2* to express the protective *APOE2* allele in *APOE4* homozygotes, aim to prevent or reverse disease progression by enhancing ApoE lipidation, increasing ApoE-associated cholesterol, and reducing Aβ levels. This approach has been effective in mouse models overexpressing mutant *APP* or *APP/PS1/APOE4* transgenes, offering a glimpse into potential therapies for altering APOE function and addressing AD pathology (Xu et al., 2015).

### 4.2 Lipid-sensitive nuclear receptors

Retinoid X receptors (RXRs), LXRs, and PPARs are all members of the nuclear receptor superfamily, and act as key regulators of lipid balance within the body and brain. They activate the genes that play critical roles in lipid metabolism (Table 3; Ma and Nelson, 2019).

#### 4.2.1 LXRs and RXRs agonists

LXRs, including LXRα and LXRβ, and RXRs, play a crucial role in lipid homeostasis by forming heterodimers and activating genes essential for lipid metabolism, with LXRβ being the dominant form in the brain, significantly higher than LXRα expression (Ma and Nelson, 2019; Gofflot et al., 2007). LXRs act as cholesterol sensors, promoting the expression of genes responsible for cholesterol efflux, such as ApoE, and can be activated alongside RXRs by their respective ligands to stimulate the transcription of *ABCA1*, *ABCG1*, and *APOE*. This activation pathway is explored in therapies aiming to correct ApoE4 structure, enhance ApoE lipidation, and facilitate Aβ clearance through strategies such as gene therapy and immunotherapy, showing promise in reversing memory deficits in AD mouse models (Olivares et al., 2015; Zelcer and Tontonoz, 2006; Liang et al., 2004; Cramer et al., 2012; Boehm-Cagan and Michaelson, 2014). LXR agonists, including T0901317 and GW3965, have been shown to increase ApoE lipidation and improve microglial association with Aβ plaques, enhancing Aβ clearance and mitigating cognitive impairments, though they also bring unwanted side effects on liver health and triglyceride production by inducing lipogenesis-related genes (Koldamova et al., 2005; Namjoshi et al., 2013; Schultz et al., 2000; Jalil et al., 2019; Cummings et al., 2016). RXRs can dimerize with themselves or with PPARs and retinoic acid receptors (RARs) in response to natural ligands, such as retinoic acid and DHA. RXRs agonists such as bexarotene and LG100268, have been found to further promote LXRs and PPAR-γ dimerization and activation, thus stimulating ApoE lipidation and ABCA1 activity, improving cognitive deficits in AD mouse models. Additionally, specific flavonoids from Sophora tonkinensis (SPF1 and SPF2) with selective RXRs agonistic activity enhanced ABCA1 upregulation and promoted ApoE lipidation. These findings suggest a potential for RXRs and LXRs agonists in AD therapy, although the therapeutic development of synthetic LXRs agonists is hampered by their peripheral side effects, highlighting the need for strategies that balance efficacy with safety (Corona et al., 2016; Boehm-Cagan and Michaelson, 2014; Tai et al., 2014; Wang et al., 2019b; Wang et al., 2019a).

#### 4.2.2 PPARγ

PPARγ stands out as the most explored PPAR target in AD research, with early studies highlighting the role of PPARγ agonists in combatting AD pathologies through anti-inflammatory actions. These include inhibiting pro-inflammatory transcription factors such as NFκB and STATs, showcasing the deep ties between lipid metabolism and the inflammatory response (Daynes and Jones, 2002; DiBattista et al., 2016). In animal studies, PPARγ agonists have been shown to mitigate Aβ pathology and enhance cognitive functions by influencing both Aβ production and clearance (Sastre et al., 2003; Pedersen et al., 2006; Mandrekar-Colucci et al., 2012; Yamanaka et al., 2012; O’Reilly and Lynch, 2012; Toledo and Inestrosa, 2010; Yu et al., 2015). However, larger human trials with PPARγ agonists, such as pioglitazone and rosiglitazone, have yielded negative outcomes (Burns et al., 2021; Gold et al., 2010; Watson et al., 2005; Tzimopoulou et al., 2010; Harrington et al., 2011), a discrepancy that might stem from these agonists’ limited ability to penetrate the BBB. This suggests that next-generation PPARγ agonists with enhanced BBB permeability could offer more effective AD interventions by targeting lipid dysregulation more efficiently.

Conversely, PPARα plays a crucial role in regulating genes crucial for fatty acid oxidation, including those for carnitine palmitoyl transferases and acyl-CoA oxidase, predominantly in astrocytes within the brain (Reddy and Hashimoto, 2001; Zhang et al., 2014; Basu-Modak et al., 1999). In the context of AD, PPARα contributes to amyloid metabolism regulation by promoting the non-amyloidogenic α-secretase activity and suppressing amyloidogenic β-secretase, offering a potential therapeutic angle for AD. Indeed, the PPARα agonist Wy-14643 has been found to alleviate AD-like pathologies and memory impairments in APP/PS1 mice, indicating its potential for therapeutic applications in AD (Zhang et al., 2015; Corbett et al., 2015; Inestrosa et al., 2013; Luo et al., 2020).

### 4.3 Other therapies affecting lipid metabolism

#### 4.3.1 High-cholesterol diet

Research has indicated that high-cholesterol diets activate astrocytes and amplify the detrimental effects of Aβ on nAChR subunits, leading to cognitive impairments associated with learning and memory (Luo et al., 2020). Elevated cholesterol levels may further impede α-secretase activity in the metabolic processing of APP and reduce the expression of α7nAChR, with AD patients showing a significant decline in these receptors’ expression (Xiu et al., 2006). Although numerous animal studies have linked HFD to impaired brain insulin signaling (Clegg et al., 2011; Carvalheira et al., 2003), few human studies have directly investigated the impact of diet on brain insulin levels. One study found that a diet high in fats and glycemic index lowers CSF insulin levels, aligning with patterns seen in AD, whereas a diet low in these components raised insulin levels in individuals with MCI to those of healthy subjects, with dietary changes in CSF insulin also correlating with shifts in CSF Aβ42 levels (Bayer-Carter et al., 2011). Systematic reviews of dietary interventions focusing on increased intake of PUFAs, nuts, and plant-based foods while reducing saturated fats, animal proteins, and refined sugars have shown that adherence to such diets is associated with improved peripheral insulin sensitivity and a reduced risk of cognitive decline and AD (Solfrizzi et al., 2017; van den Brink et al., 2019; Pistollato et al., 2018).

Epidemiological studies have suggested that a higher intake of ω-3 PUFAs, particularly DHA and EPA, is associated with a reduced risk of AD, whereas a lower intake increases this risk (Morris et al., 2003; Zhang et al., 2016). DHA and EPA are believed to diminish Aβ production and augment its degradation, modulating the brain’s inflammatory response to Aβ, and acting as activators of RXRs and PPARs (de Urquiza et al., 2000; Forman et al., 1997; Casali et al., 2015). The supplementation of ω-3 FA in patients with MCI has been shown to enhance the macrophage levels of specialized pro-resolving mediators (SPMs) like RvD1, increase Aβ phagocytosis, and lower cytokine expression, suggesting that the resolution of neuroinflammation via SPMs could mediate some of the beneficial effects of ω-3 FA (Fiala et al., 2015).

Ketogenic diets (KDs), high in fat and low in carbohydrates, produce ketone bodies such as β-hydroxybutyrate and acetoacetate, which serve as alternative fuel sources for the brain, particularly in AD, where glucose metabolism is compromised early in the disease’s development (Martin et al., 2006; Mosconi et al., 2008; Cunnane et al., 2011). KDs have shown promise in attenuating Aβ pathology and enhancing metabolic and cognitive functions in animal models of aging and AD, with clinical trials in early-stage AD patients demonstrating cognitive improvements (Cunnane et al., 2011; Newman et al., 2017; Kimoto et al., 2017; Henderson and Poirier, 2011; Henderson et al., 2009; Kashiwaya et al., 2000). Moreover, ketone bodies may promote neuronal growth and survival and potentially upregulate mitochondrial biogenesis, thus improving oxidative phosphorylation and ATP production in the brain (Kashiwaya et al., 2000; Bough et al., 2006). However, the efficacy of KDs against AD may vary based on the individual metabolic status and lipid profiles, highlighting the need for further research to determine the long-term applicability of KDs in AD patient care.

#### 4.3.2 Statins

Statin therapy is known to decrease cholesterol levels in the brain membranes, and further exhibits anti-inflammatory, antioxidant, and neuroprotective properties. Although preclinical, retrospective/prospective, and observational studies have suggested that statins may offer protective benefits against AD development, randomized clinical trials have not confirmed the beneficial effects of statins in patients (Dai et al., 2021; Geifman et al., 2017; Li G. et al., 2010; Sano et al., 2011; Feldman et al., 2010; Li et al., 2017). The potential protective mechanism of statins is thought to be derived largely from lowering circulating cholesterol levels, which positively affects the cardiovascular and cerebrovascular systems and diminishes vascular inflammation, indirectly impacting AD risk (Schultz et al., 2018). Additionally, the influence of statins in reducing the risk of AD may also be related to their effects on vascular dementia, which is frequently mistaken for AD. Recent research has suggested that statin therapy could be particularly advantageous for individuals with the ApoE4 genotype, aligning with the role of ApoE4 in disrupting both the central and systemic cholesterol balance. Beyond cholesterol regulation, statins have been proposed to mitigate neuroinflammatory processes that contribute to neurodegeneration (Geifman et al., 2017; Dagliati et al., 2021; McFarland et al., 2018).

#### 4.3.3 Gut microbiota modulation

Probiotics have recently been recognized as an effective and safe method for altering the composition of the gut microbiota and enhancing overall health through mechanisms that are still being explored. In particular, chronic dietary supplementation with a multi-strain probiotic formulation, SLAB51, showed promising results in 3xTg-AD mice. This treatment adjusted the gut microbiota, improved glucose metabolism, reduced inflammatory and oxidative states, partially restored impaired neuronal proteolysis, and diminished Aβ and tau aggregates, leading to enhanced cognitive functions and a delay in AD progression (Bonfili et al., 2017; Bonfili et al., 2018; Bonfili et al., 2020). Probiotic administration has also been found to suppress cholesterol biosynthesis in mice with AD via pathways involving sterol regulatory element-binding protein 1c and LXRs, along with increased brain expression of cholesterol 24S-hydroxylase. These findings suggest that modulating the microbiota with probiotics can beneficially alter the lipid composition in AD mice, highlighting arachidonic acid as a key metabolite linking changes in the lipid profile induced by probiotics to insulin sensitivity and inflammation.

Short-chain fatty acids (SCFAs), which include acetate, butyrate, and propionate, are produced by colonic bacteria through the anaerobic fermentation of dietary fibers and undigested carbohydrates, and range from one to six carbon atoms in length (Tan et al., 2014). These SCFAs can cross the BBB or influence the brain via the gut-brain axis, with butyrate offering neuroprotective benefits as a histone deacetylase inhibitor, affecting G protein-coupled receptor signaling pathways, and exhibiting anti-inflammatory effects (Braniste et al., 2014). Butyrate has further been shown to enhance hippocampal histone acetylation and elevate the expression of genes related to learning (Bonfili et al., 2022). In AD mouse models, sodium butyrate treatment improved learning and memory functions, reduced amyloid plaque buildup, and restored dendritic spine density in the hippocampus (Govindarajan et al., 2011; Ricobaraza et al., 2012). Additionally, acetate has been observed to modulate microglial activity and decrease BBB permeability (Bienenstock et al., 2015), and is reduced in Drosophila models of AD (Kong et al., 2018), suggesting a multifaceted role for SCFAs in AD management and progression.

Taken together, summarizing the clinical trials targeting lipid metabolism in MCI or AD in a table will enhance the clarity and accessibility of this information (Table 4).

**TABLE 4:** Summarizes several clinical trials targeting lipid metabolism in MCI or AD.

Trial Name	Phase	Objective	Sample Size	Intervention	Outcome Measures	Status	ID
EPOCH Study	Phase 2	Assess the efficacy of a lipid-lowering agent in reducing AD symptoms	150	Statin Therapy (Atorvastatin 80 mg/day)	Cognitive function, lipid levels	Completed	NCT02489044
Ketogenic Diet in AD	Phase 1/2	Evaluate the safety and efficacy of a ketogenic diet in AD patients	60	Ketogenic Diet (High-fat, low-carbohydrate diet)	Cognitive function, metabolic parameters	Recruiting	NCT03767573
ApoE4 Lipidation Enhancer	Phase 1	Investigate the effects of enhancing ApoE lipidation on cognitive function in AD	40	ApoE4 Lipidation Enhancer (CS-6253 5 mg/kg)	Cognitive function, Aβ levels	Completed	NCT03003496
AAV-APOE2 Gene Therapy	Phase 1/2	Investigate the safety and efficacy of delivering the protective ApoE2 allele to ApoE4 carriers, aiming to enhance lipid metabolism and reduce Aβ burden	15	LX1001 (1.4 x 10^10 gc/mL CSF of LX1001)	APOE protein levels and Alzheimer’s biomarkers in the cerebrospinal fluid	Active, not recruiting	NCT03634007
NE3107 in AD	Phase 3	Determine the efficacy of NE3107, a novel compound targeting lipid metabolism, in AD	240	NE3107 20 mg/day	Cognitive function, inflammation markers	Active, not recruiting	NCT05064712
LXR and RXR Agonists	Phase 3	Determine the safety and efficacy of renal denervation in patients with uncontrolled hypertension	530	RXR agonist (Bexarotene)	Changes in office blood pressure and safety assessments	Completed	NCT01782742
PPAR Agonist in AD	Phase 2	Study the impact of PPAR agonists on lipid metabolism and cognitive function in AD	80	PPAR Agonist (Pioglitazone 30 mg/day)	Cognitive function, lipid metabolism parameters	Recruiting	NCT03693135
TOMMORROW Study	Phase 3	To qualify a biomarker risk algorithm for predicting the development of Mild Cognitive Impairment due to Alzheimer’s Disease (MCI-AD) and to evaluate the efficacy of pioglitazone in delaying the onset of MCI-AD in cognitively normal participants at high risk	3494	PPAR Agonists (pioglitazone 0.8 mg sustained release)	Diagnosis of MCI-AD	Terminated early due to lack of efficacy in preventing the onset of MCI-AD	NCT01931566
Fish Oil Supplementation in MCI	Phase 2	Assess the benefits of omega-3 fatty acids in cognitive decline	120	Fish Oil Supplementation (DHA 1 g/day + EPA 1 g/day)	Cognitive function, lipid levels	Completed	NCT02578523

MCI, mild cognitive impairment; AD, Alzheimer’s disease.

## 5 Summary

Extensive clinical research has shown significant changes in lipid composition and metabolism to be at the core of AD development and progression, with alterations often appearing at disease onset. This evidence points to lipid imbalance as a potential early trigger of AD, likely through its interaction with amyloid buildup, synapse formation, and energy metabolism, underscoring the need for timely lipid-focused intervention strategies.

As AD advances, lipid imbalances not only persist but also intensify, potentially driving a harmful cycle that exacerbates neuroinflammation, energy failure, and oxidative damage, thus accelerating neuronal loss and cognitive decline. This outlook suggests that dysregulated lipid metabolism plays a central role in bridging the gap between reduced cellular energy and ongoing brain inflammation, which are the key features of AD. Moreover, the complex interplay between lipid transport, Aβ, and ApoE underscores the potential of lipid metabolism as a critical junction for both direct and indirect pathways influencing AD pathology.

Insights from preclinical successes and shortcomings of clinical trials targeting lipid metabolism in AD have highlighted the influence of genetic factors, particularly the ApoE genotype, on treatment outcomes. Treatments aimed at modulating lipid metabolism appear to be more promising for AD prevention, especially among individuals carrying the ApoE4 allele, who constitute a significant proportion of both the general and AD-affected populations. Future therapeutic developments must consider the need to target the brain while minimizing side effects on peripheral organs, such as the liver. The collective findings of decades of research emphasize the importance of tailoring lipid metabolism interventions to individual risk profiles, including genetic and lifestyle factors, to effectively combat AD. Overall, adapting therapeutic approaches to address the evolving landscape of lipid metabolism across all stages of AD could enhance treatment efficacy.
